# Characteristics of the *fads*2 gene promoter in marine teleost *Epinephelus coioides* and role of Sp1-binding site in determining promoter activity

**DOI:** 10.1038/s41598-018-23668-w

**Published:** 2018-03-28

**Authors:** Dizhi Xie, Zhixiang Fu, Shuqi Wang, Cuihong You, Óscar Monroig, Douglas R. Tocher, Yuanyou Li

**Affiliations:** 1 College of Marine Sciences, South China Agricultural University, Guangzhou, 510842 China; 2 Guangdong Provincial Key Laboratory of Marine Biotechnology, Shantou University, Shantou, 515063 China; 3 Institute of Aquaculture, School of Natural Sciences, University of Stirling, Stirling, FK9 4LA Scotland UK

## Abstract

Δ6 fatty acyl desaturase (Fads2) is a rate-limiting enzyme in long-chain polyunsaturated fatty acids (LC-PUFA) biosynthesis. Comparative analysis of gene promoters of Fads2 between salmonids and carnivorous marine fish suggested that the lack of binding site for stimulatory protein 1 (Sp1) was responsible for the low expression of *fads*2 gene of carnivorous marine species. To confirm this speculation, the *fads*2 candidate promoter (2646 bp) was cloned from carnivorous marine teleost *Epinephelus coioides*, and 330 bp core regulatory region was identified. Several binding sites for transcriptional factors such as nuclear factor 1, nuclear factor Y, sterol regulatory element and hepatocyte nuclear factor 4γ were identified, while that for Sp1 was shown to be absent in the promoter by both bioinformatic analysis and site-directed mutation. Moreover, after the Sp1-binding site from the *fads*2 promoter of herbivorous *Siganus canaliculatus*, the first marine teleost demonstrated to have LC-PUFA biosynthetic ability, was inserted into the corresponding region of *E*. *coioides fads*2 promoter, activity was significantly increased. The results provided direct data for the importance of the Sp1-binding site in determining *fads*2 promoter activity, and indicated that its lack may be a reason for low expression of *fads*2 and poor LC-PUFA biosynthetic ability in *E*. *coioides*.

## Introduction

Long-chain (C_20–24_) polyunsaturated fatty acids (LC-PUFA) are essential fatty acids (EFA) for both human and marine teleosts. In particular, eicosapentaenoic acid (EPA, 20:5n–3) and docosahexaenoic acid (DHA, 22:6n–3) play important roles in maintaining normal development of the nervous system and sensory organs^1,2^, promoting cardiovascular health and immune function^3,4^, and involving in the regulation of lipid metabolism^5,6^. Fish, especially marine species, are the primary source of LC-PUFA in the human diet. However, with overfishing and the degradation of the marine environment, natural wild fishery stocks have reduced sharply. Thus, the declining capture fisheries has turned attention to farmed marine fish as the major source of LC-PUFA. Fish oil (FO), which is rich in LC-PUFA, was traditionally utilised for meeting the requirement of EFA in farmed marine fish systems. However, the limited availability of FO is a major limitation for the development of marine fish aquaculture^7^. Therefore, much attention has been given to seeking the sustainable alternatives, especially vegetable oils (VO), which are rich in linolenic acid (LNA; 183n–3) and linoleic acid (LA;18:2n–6), but devoid of LC-PUFA. Unfortunately, due to most farmed marine fish having only limited capability or inability for converting LNA and LA to LC-PUFA, dietary VO usually have negative effects on the contents of n–3 LC-PUFA of farmed fish^8^. Thus, much attention has been focused on elucidating the regulatory mechanisms of LC-PUFA biosynthesis, in order to maximize endogenous production in marine fish.

The LC-PUFA biosynthesis pathway involves consecutive desaturation and elongation steps from LNA and LA catalyzed by fatty acyl desaturase (Fads) and elongation of very long-chain fatty acids (Elovl) enzymes^9^. Consequently, the LC-PUFA biosynthetic ability of fish depends on the expression levels and activities of these key enzymes. Among them, Δ6 Fad (Fads2) is the rate-limiting enzyme, which is responsible for catalyzing the first step in the LC-PUFA biosynthetic pathway, and also involved in DHA synthesis from EPA, and has been commonly regarded as an indicator of LC-PUFA biosynthetic ability in fish^10,11^. Although the activity and nutritional regulation of Fads2 have been investigated in several fish species^12–16^, the molecular mechanisms underlying their regulation remains largely unknown. The comparative analysis of *fads*2 gene promoter between *Salmo salar* (with LC-PUFA biosynthetic ability) and *Gadus morhua* (a carnivorous marine teleost with very limited LC-PUFA biosynthesis) suggested that low expression of *fads*2 in the latter could be attributed, at least partly, to the lack of a binding site of stimulatory protein 1 (Sp1) in the *fads*2 promoter^17^. Similarly, the Sp1 binding site was not found in the *fads*2 promoter of the carnivorous marine teleost *Dicentrarchus labrax* either, and its *fads*2 promoter activity was weaker than that in *Oncorhynchus mykiss*^11^. Therefore, it was speculated that the Sp1 site may play an important role in determining the *fads*2 promoter activity and LC-PUFA biosynthetic ability in fish^11,17^. However, there was no direct evidence to support such a deduction.

Rabbitfish *Siganus canaliculatus* is a herbivorous marine teleost widespread along the Indo-West Pacific coast and cultured in southeastern Asia including China due to its popularity in markets. It is noteworthy that *S*. *canaliculatus* was the first marine teleost demonstrated to have the ability of LC-PUFA biosynthesis, with key enzymes including Fads2, and Elovl4 and Elovl5 elongases required for LC-PUFA biosynthesis being identified and characterized in this species^14,18–20^. Moreover, sequence analysis identified an Sp1 binding site in the Δ6/Δ5 *Fad* promoter of *S*. *canaliculatus* (unpublished data). So, *S*. *canaliculatus* provides us a favourable model for investigating the regulatory mechanisms of LC-PUFA biosynthesis in teleosts. On the other hand, the grouper *Epinephelus coioides* is a typical carnivorous marine teleost, which has been widely cultured in coastal areas of southeastern Asia including China for its fast growth performance and huge economic value^12^. Moreover, the genes encoding Fads2 of this species have been cloned and functionally characterized in an exogenous yeast system^12^, which showed that the Fads2 had low enzymatic activity in converting LNA and LA to 18:4n–3 and 18:3n–6, respectively. The results were consistent with a recent feeding trial, which showed that juvenile *E*. *coioides* has poor LC-PUFA biosynthetic capacity^21^. In order to investigate the underlying reasons, the present study was focused on clarifying the importance of the Sp1 binding site in determining the transcriptional activity of the *fads*2 promoter, as speculated in marine carnivorous *G*. *morhua* and *D*. *labrax*. First, the candidate promoter of *E*. *coioides fads*2 gene was cloned and functionally analyzed. Then, targeted mutation of potential transcriptional factor (TF) binding sites was performed to identify key elements in the *fad* promoter. Moreover, the role of the Sp1 binding site in the *fads*2 promoter was clarified. The results will be helpful for identifying the reasons underpinning the low LC-PUFA biosynthetic ability of *E*. *coioides*, and will provide novel insights into the regulatory mechanisms of LC-PUFA biosynthesis in vertebrates.

## Results

### Tissue distribution of *E*. *coioides fads*2 mRNA

**Figure 1:**
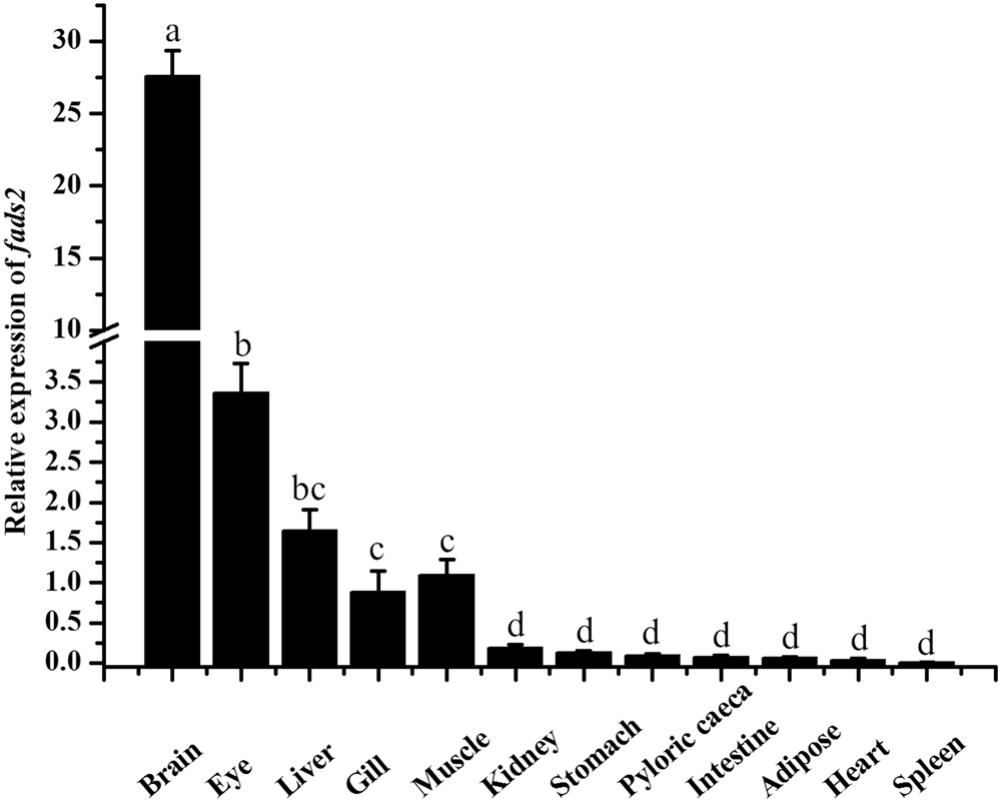
Tissue distribution of *fads*2 mRNA in *Epinephelus coioides* examined by qPCR. Relative expression of *fads*2 were quantified for each transcript and were normalized with β-actin by 2^−ΔΔCt^ method. Results are means ± SEM (n = 6), bars without sharing a common letter indicated significant differences (*P* < 0.05) among tissues as determined by one-way ANOVA followed by Tukey’s multiple comparison test.

The tissue distributions of *E*. *coioides fads*2 were determined by qPCR. The highest expression of *fads*2 were detected in the brain, followed by the eyes, liver, muscle and gill, and relatively low expression of *fads*2 were observed in other tissues (Fig. 1). Moreover, the expression of *fads*2 in the brain was much higher than that in other tissues.

### Organization of *E*. *coioides fads*2 promoter

**Figure 2:**
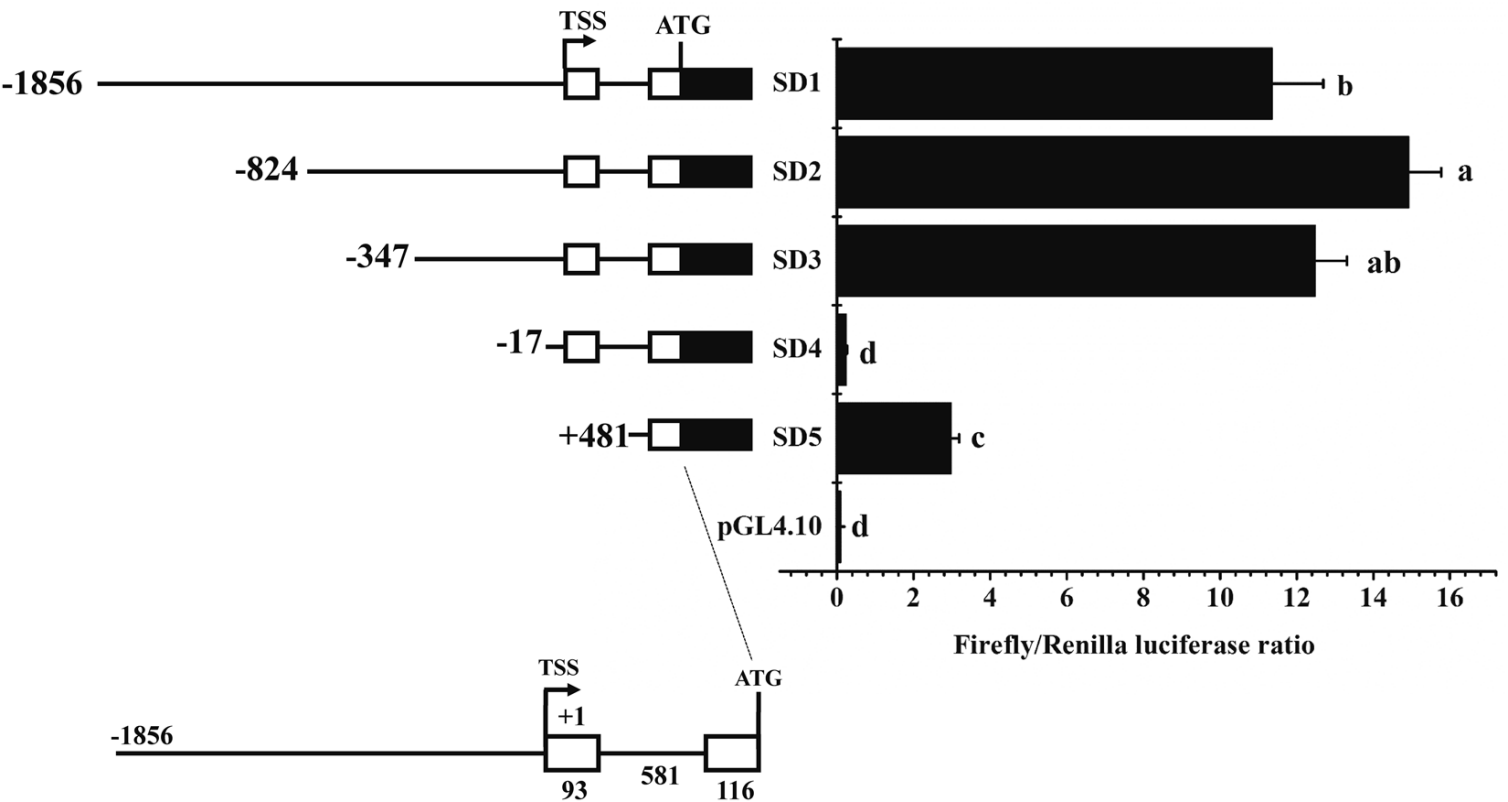
Structure and progressive deletion analysis of *Epinephelus coioides fads*2 promoter. 5′ deletion constructs are shown on the upper left, and the structure of of *fads*2 promoter is showed on the lower left. Non-coding exons are indicated with open boxes, and luciferase coding frame by closed boxes. Sequence is numbered relative to the first base of the transcription start site (TSS), assumed to be the first base of the 5′ non-coding exon. Numbers refer to exons (open boxes) and intron (line) sizes in base pair. Promoter activity of constructs is represented on the right with the values representing normalised activity (Firefly luciferase/Renilla luciferase). Bars without sharing a common letter indicated significant differences (*P* < 0.05) among deletions determined by one-way ANOVA followed by Tukey’s multiple comparison test.

The candidate promoter of *fads*2 cloned in this study was 2646 bp in length, including 1856 bp upstream nontranscribed sequence and 790 bp 5′-UTR sequence of *fads*2 gene. The 790 bp 5′-UTR sequence consists of exon 1, intron 1 and partial sequence of exon 2, the schematic diagram of *fads*2 promoter structure was shown in Fig. 2 and Supplementary Fig. S1.

### Determination of core regulatory region of *E*. *coioides fads*2 promoter

To determine the core regulatory region of the *fads*2 promoter, full length of candidate promoter and its 5′ serial truncations were fused to a promoterless luciferase reporter vector pGL4.10 [luc2] and tested for ability to mediate transcription. Transfection of Tilapia HepaT cells with progressive deletions showed that maximal promoter activity appeared when up to 824 nucleotides upstream of the transcription start site (TSS) were included in the construct (SD2). The activity of construct SD3 decreased but showed no significant difference with SD2. However, when 5′ truncation came to 17 bp upstream of TSS (SD4), the activity of promoter decreased to the level as negative control. The results indicated that the region between −347 to −17 may contain some important regulatory elements (Fig. 2). Therefore, the region between −347 to −17 was identified as the core regulatory region of the *E*. *coioides fads*2 promoter, and the sequence of construct SD3 (from −347 to +116) was used for further functional analysis.

### Identification of cis-acting elements in core regulatory region of promoter

**Figure 3:**
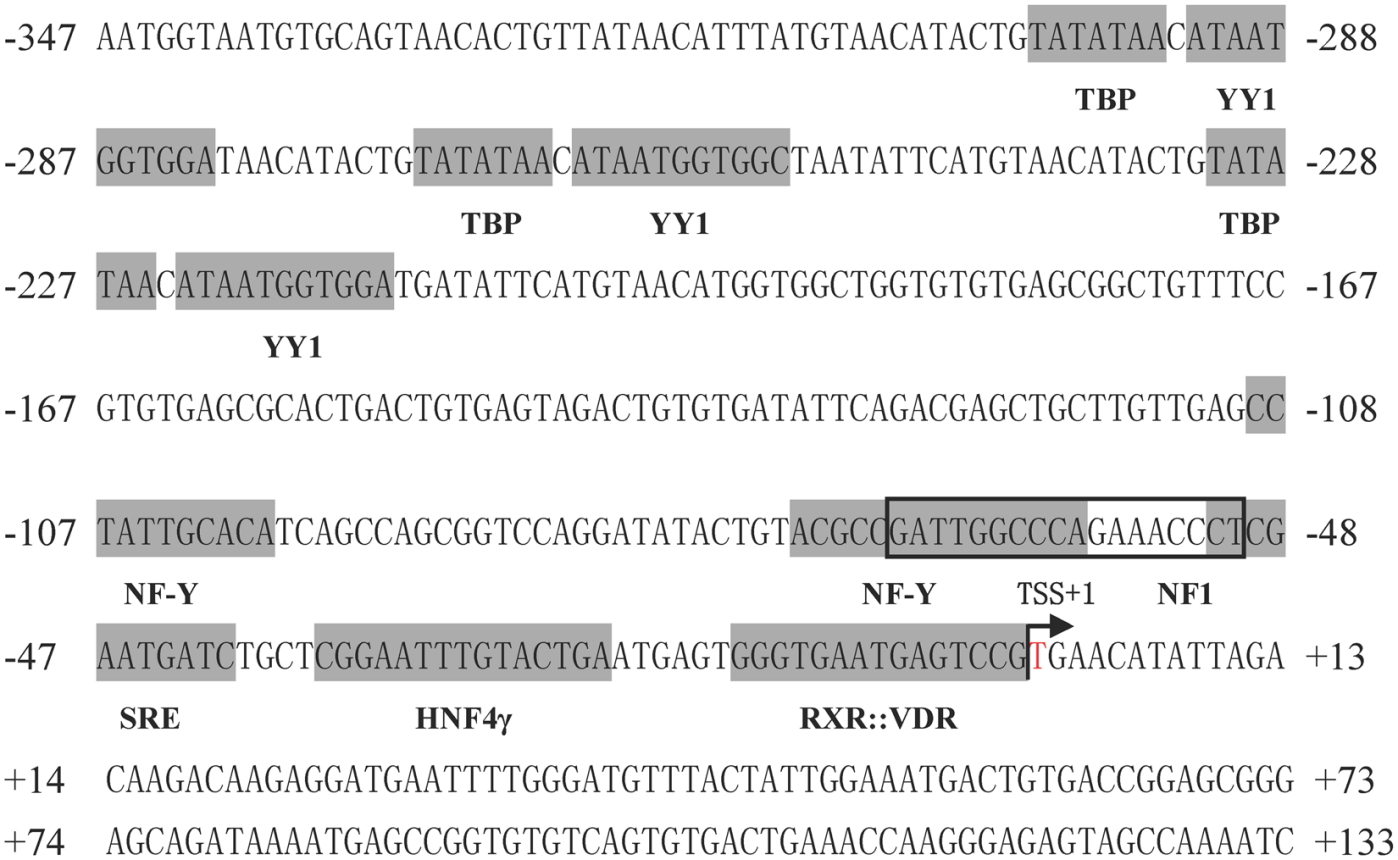
The nucleotide sequence and predicted binding sites for transcription factors in the core region of *Epinephelus coioides fads*2 promoter. Numbers are given relative to the first base of the transcription start site (TSS). Potential transcription binding motifs are marked in grey or open boxes for NF1. Details for the name of transcription factors can be found in the text.

**Figure 4:**
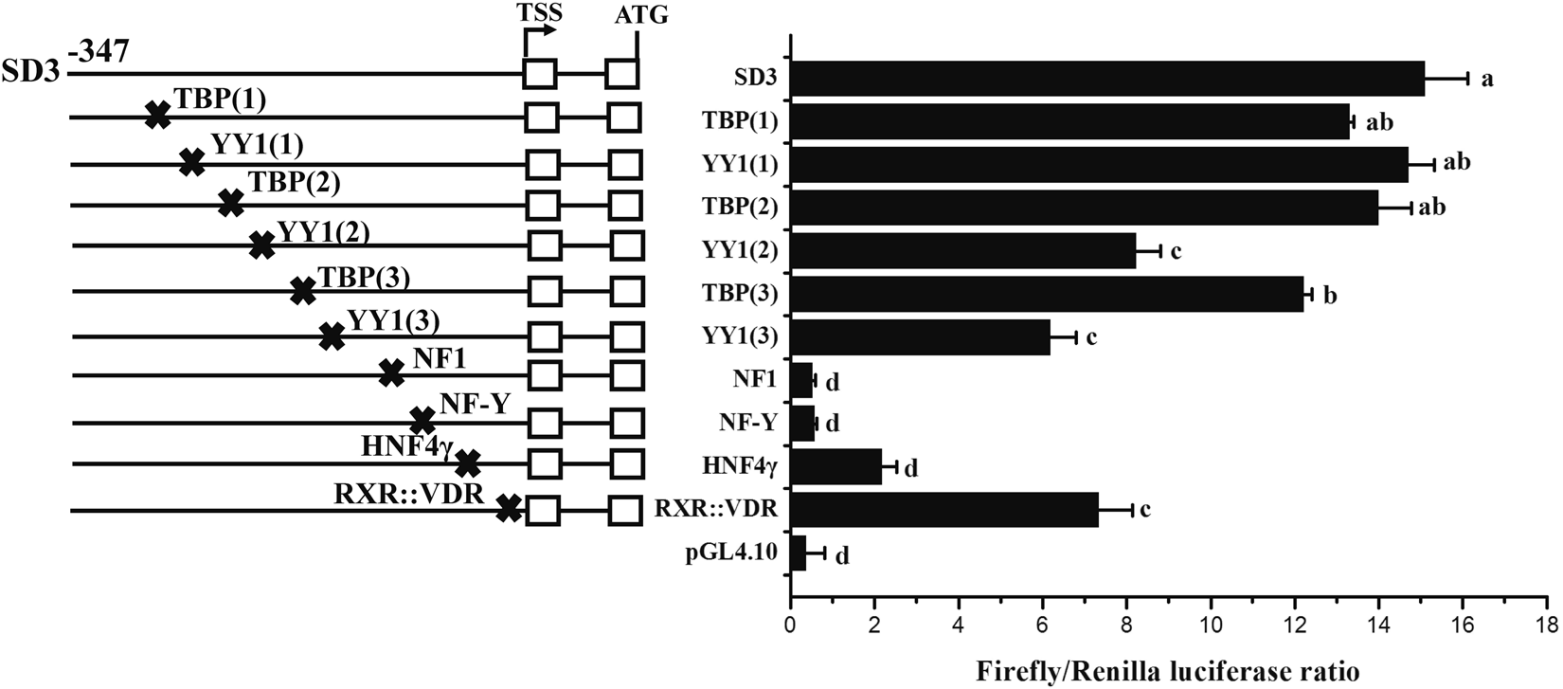
Effects of transcription factor mutations on *Epinephelus coioides fads*2 promoter activity. Mutations of promoter deletion on SD3 (−347 to ATG) were generated according to *in silico* prediction, and the effects of mutation on promoter activity were compared with wild type (SD3). Promoter activity of constructs is represented on the right with the values representing normalized activity (Firefly luciferase/Renilla luciferase). Bars without sharing a common letter indicated significant differences (*P* < 0.05) among deletions determined by one-way ANOVA followed by Tukey’s multiple comparison test.

The core regulatory region of *E*. *coioides fads*2 promoter was subjected to further *in silico* analysis with TRANSFAC^®^, MatInspector^®^ and JASPAR^®^, and several potential binding sites for TF such as TBP, YY1, NF-Y, NF1, HNF4γ and RXR::VDR were predicted (Fig. 3). After these binding sites were respectively site-directedly mutated, their effects on promoter activity were investigated in HepaT cells transfected with each mutant. The results showed that mutation of binding sites for NF1, NF-Y, HNF4γ, RXR::VDR, TBP (one of three) and YY1(two of three) caused significant reduction of promoter activity (Fig. 4), indicating these sites are key elements in *fads*2 promoter of *E*. *coioides*.

### Effect of bases replacement in Sp1 binding site on promoter activity

**Figure 5:**
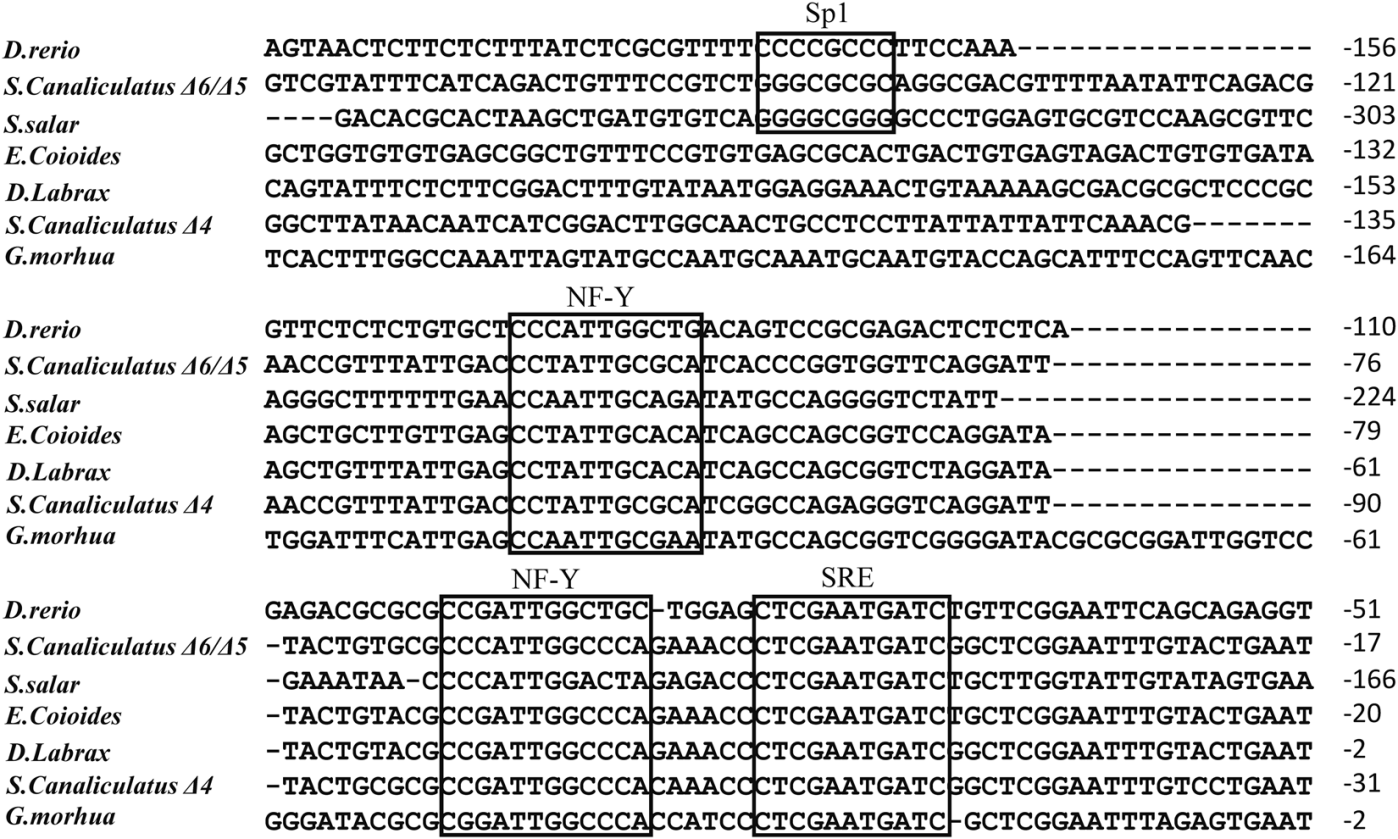
Alignment of *fads*2 promoters among *Epinephelus coioides* and other fish species. The numbers indicate sequence positions relative to possible transcription start site. Binding sites for Sp1, NF-Y and SREBP are shown in boxes based on previous studies^11,17,32,33^.

**Figure 6:**
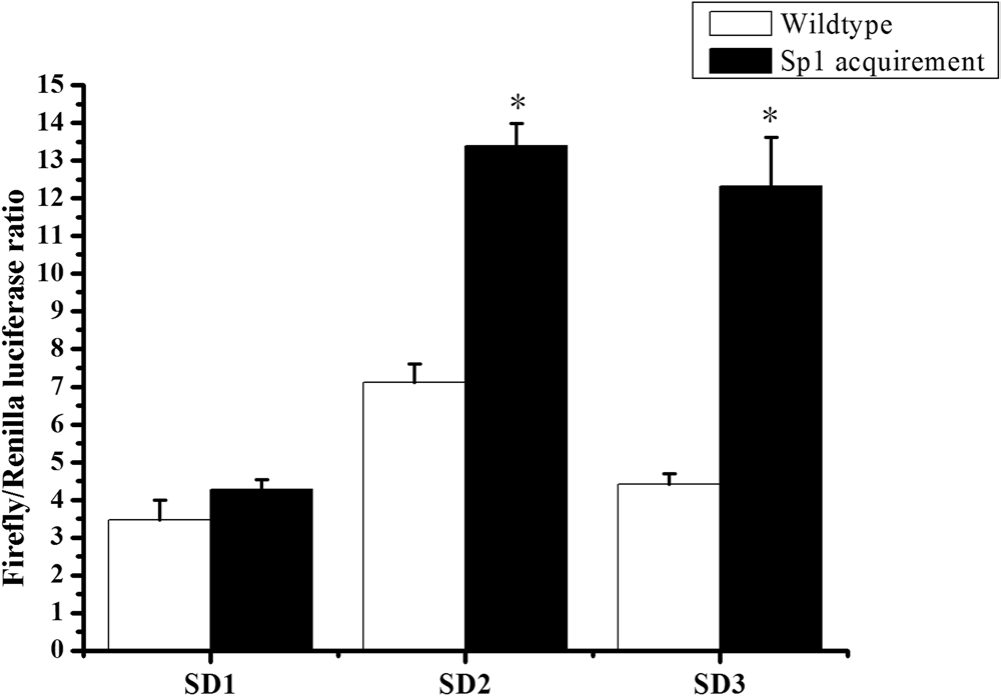
Mutation of Sp1 binding site acquirement on *Epinephelus coioides fads*2 promoter with different length. Effects of Sp1 site replacement on each construct (SD1, SD2 and SD3) were compared with wildtype. *Represents significant difference between Sp1 replacements and wildtypes, respectively.

Alignment of seven *fads*2 promoters from six fish species showed that binding sites for NF-Y, NF-Y and SRE exist in all species examined, indicating they are conserved elements in *fads*2 promoter. However, the Sp1 binding site was just found in *fads*2 of *Danio rerio*, *S*. *salar*, and *S*. *canaliculatus* (Δ6/Δ5 *fad*), but absent in carnivorous marine teleost such as *E*. *coioides*, *D*. *labrax* and cod *G*. *morhua* (Fig. 5). These characteristics suggested that Sp1 may play an important role in determining the transcription activity of *fads*2, which further affect the LC-PUFA biosynthetic ability of fish. In order to confirm this speculation, the corresponding sequence in *fads*2 promoter of *E*. *coioides* was mutated into the same sequence of Sp1 binding site in *fads*2 (Δ6/Δ5 *fad*) promoter of *S*. *canaliculatus*. Dual luciferase assay showed that the activity of *E*. *coioides fads2* promoter with Sp1 binding site was significantly increased as showed in constructs SD2 and SD3 (Fig. 6), indicating that lack of Sp1 binding site in *E*. *coioides fads*2 promoter may be an important reason for low activity.

## Discussion

The present study aimed to gain insights into the mechanisms underlying transcriptional regulation of LC-PUFA biosynthesis in *E*. *coioides*. To this end, the tissue distribution and transcriptional regulation of *fads2* were investigated. The rate-limiting enzyme *fads2* had been cloned from some fish species, and functionally characterized in exogenous expression system^10,12,13,15,16,19^, and its tissue distribution was also investigated in a variety of fish species^13,16,17,22–24^. In the present study, the highest expression of *E*. *coioides fads*2 mRNA in the brain was consistent with the situations in other carnivorous marine fish species like *G*. *morhua*, *Larimichthys crocea* and *Nibea coibor*^16,25,26^. In contrast, higher *fads*2 mRNA expression in liver was detected in some species with LC-PUFA biosynthetic ability, such as freshwater fish, salmonids and herbivorous marine fish^18,22,27,28^. Furthermore, liver has been regarded the primary tissue for LC-PUFA synthesis^29^. These data indicate that the low level of hepatic *fads*2 transcripts in carnivorous marine fish, like *E*. *coioides*, may correlate with their limited LC-PUFA biosynthetic ability^30^.

Generally, the level of gene transcription in eukaryotic cells is greatly dependent on the binding of RNA polymerase and transcription factors to specific sequences in gene promoters^31^. Thus, the activity and integrity of a promoter can influence gene transcription. The core region of the *E*. *coioides fads*2 promoter was −347 bp to −17 bp, which included NF1, NF-Y, SRE, HNF4γ and RXR::VDR cis-elements. Comparing *fads*2 promoter regions among *E*. *coioides*, *G*. *morhua*^17^, *S*. *salar*^17^, *D*. *labrax*^11^, *S*. *canaliculatu*^32^ and *D*. *rerio*^33^, the highly conserved NF-Y and SRE elements were shown in all examined species. In addition to the conserved NF-Y and SRE elements, *E*. *coioides fads*2 promoter shared the same binding site for NF1 and RXR::VDR as in *S*. *canaliculatu*, *H*. *sapiens* and *D*. *rerio fads*2 promoters^32–34^, respectively. Site directed mutagenesis revealed that cis-elements such as NF-Y, NF1 and RXR::VDR were essential in driving the activity of the *E*. *coioides fads*2 promoter, which was a consistent with the effects of these mutations on the *fads*2 promoter activity of *S*. *salar*^17^, *S*. *canaliculatus*^32^ and *H*. *sapiens*^34^.

Studies on mammalian *Fads*2 promoter have shown that transcription factors SREBP binding to SRE cis-elements usually requires the presence of co-activators like NF-Y and/or Sp1 sites^34–36^. The presence of adjacent NF-Y directly determines the binding of SRE and SREBP, which acts as a natural ligands to stimulate the basal *fads2* gene transcription^35–37^. Although SREBPs have not been well characterised in fish species until now, SREBP-1 was involved in up-regulating the activity of *fads*2 promoter by 1.58-fold, 4.57-fold and 1.59-fold in *O*. *mykiss*, Japanese seabass (*Lateolabrax japonicus*) and large yellow croaker (*Larimichthys crocea*), respectively^38^. Those results indicated that the conserved SRE element was also essential in driving *fads*2 promoter activity in fish.

Apart from these conserved elements above, like NF-Y and SRE, there were several different cis-elements in the *fads*2 core promoters of the different fish species. For example, the C/EBPα element was predicted in the promoter region of *G*. *morhua* and *S*. *canaliculatu*^17,32^, PPARα binding element was discovered in *D*. *rerio fads*2 promoter^33^, HNF4α site was discovered in *S*. *canaliculatu fads*2 (Δ4 Fad) promoter, and the HNF4γ binding sites influenced the promoter activity of *E*. *coioides fads*2. Whether HNF4γ is involved in the regulation of *fads*2 expression has not been clarified fully so far, but it can combine with HNF4α, a regulator in stimulating the *fads*2 gene transcription, to regulate gene expression^32,39^, and thus HNF4γ is likely to be involved in the regulation of the *fads*2 gene in fish. Interestingly, Sp1 elements only appear in the *fads*2 promoter regions of some fish species with LC-PUFA biosynthetic ability, such as *S*. *salar*^17^, *D*. *rerio* and *S*. *canaliculatu*^32^. However, the carnivorous marine fish species, like *G*. *morhua*, *D*. *labrax*, *L*. *japonicus*, *L*. *crocea* and *E*. *coioides*, which were shown to lack of the Sp1 elements^11,17,38^.

Sp1 has been shown to contribute with NF-Y transcription factors to the regulation by SREBP of the expression of several genes involved in cholesterogenic and lipogenic pathways^35,36^. The lack of an Sp1 binding site in carnivorous marine fish may, to some extent, account for their low *fads*2 gene expression. In comparison to salmonids, *G*. *morhua*, and *D*. *labrax fads*2 gene promoters without an Sp1 site showed lower activity, and thus it is speculated that the low expression of *fads*2 can be attributed to their incomplete and less active promoter^11,17^. Recently, the Sp1 binding site was detected in mammalian FADS and ELOVL7 promoters, and Sp1 up-regulated the expression of Fads2 and Elovl7^40,41^. These results suggested that the lack of Sp1 binding site maybe a reason leading to the low promoter activity of *fads*2, and thus result in low hepatic *fads*2 expression in carnivorous marine teleost, like *E*. *coioides*.

In the present study, the activity of *E*. *coioides fads*2 promoter with the Sp1 binding site mutations was significantly increased. The result provided direct evidence that Sp1 plays an important role in determining *fads*2 promoter activity. This was consistent with the hypothesis that the lack of a Sp1 binding site in the *E*. *coioides fads*2 promoter is a reason leading to low gene expression of *fads*2, and thus result in poor LC-PUFA biosynthetic ability in *E*. *coioides*. Interestingly, the addition of the Sp1 binding site significantly increased the activity of SD2 and SD3, while the activity of SD1 did not show a significant increase upon the same treatment. This may be attribute to the existence of some inhibitory elements located between SD1 and SD2, which requires to be investigated further.

In summary, high expression of *E*. *coioides fads*2 was detected in brain, followed by eyes, liver, muscle and gill. A sequence of 2646 bp candidate promoter of *fads*2 gene was cloned and functionally characterized, and its core regulatory region ranging from −347 bp to −17 bp identified. Binding sites for NF1, NF-Y, HNF4γ and RXR::VDR were identified as key elements in the *E*. *coioides fads*2 promoter. The lack of an Sp1 binding site in *fads*2 promoter was proved to be at least partly responsible for the low expression of hepatic *fads*2 mRNA and poor LC-PUFA biosynthetic capacity of *E*. *coioides*.

## Materials and Methods

### Ethics statement

In present study, all animal experiments were done in accordance to the National Institutes of Health guide for the care and use of Laboratory animals (NIH Publications No. 8023, revised 1978) and approved by the Animal Care and Use Committee of South China Agricultural University (Guangdong, China). All surgery was performed under 0.01% 2-phenoxyethanol (Sigma-Aldrich, St. Louis, MO, USA) anesthesia, and all efforts were made to minimize suffering of fish.

### Animals and tissue collection

*E*. *coioides* juveniles were bought from a local aquafarm in Fujian, China. After they were reared in floating cages (1 m × 1 m × 1.5 m) at Nan Ao Marine biology station (NAMBS) of Shantou University and fed a formula feed with 50% crude protein and 10% crude lipid for two weeks, six individuals (body weight ~24 g) were anaesthetized with 0.1% 3-amino benzoate methane sulphonate (MS-222; Sigma, Japan). Tissue samples including brain, eye, gill, liver, intestine, kidney, heart, muscle, spleen, stomach, pyloric caecum and fat (adipose) were collected and immediately frozen in liquid nitrogen, then stored at −80 °C until further use for the analysis of *fads2* transcripts distribution, or genomic DNA isolation.

### RNA isolation and qPCR for measuring the tissue-specific distribution of fads2 mRNA in *E*. *coioides*

Tissue distribution of *fads*2 mRNA was determined by quantitative real time PCR (qPCR). Total RNA was respectively extracted from the above tissue samples using TRIzol^®^ Reagent (Invitrogen, USA) according to manufacturer’s protocol, and 1 μg of total RNA was reverse-transcribed into cDNA using random hexamers (Tiangen, China). Gene-specific primers (*fads2*-F CCCTATCATCACCAACACCAGT, *fads2*-R GGGAATGTAACAGCACAGGTAG; *β-actin*-F TGTCTTTCCCTCCATCGTCGG, *β-actin*-R CCCAGTTGGTCACAATACCGT) were used for qPCR analysis. qPCR was carried out with an initial activation step at 95 °C for 5 min, followed by 40 cycles: 10 s at 95 °C, 20 s at 60 °C and 20 s at 72 °C. After the amplification phase, a dissociation curve of 0.5 °C increments from 65 °C to 95 °C was performed, enabling confirmation of the amplification of a single product in each reaction. The relative expression of *fads*2 were normalized with *β-actin* expression calculated by the 2^−ΔΔCt^ method^42^.

### Cloning of *fads*2 promoter and construction of deletion mutants

Genomic DNA was extracted from the liver tissue of *E*. *coioides* as described previously^32^ and used as template for candidate promoter cloning. The sequence upstream of *fads*2 gene was obtained from the genomic sequencing data of *E*. *coioides*. For identifying the core region within 5′ flanking sequence of the E. coioides *fads*2, one of the forward primers (SD1-F, SD2-F, SD3-F, SD4-F, SD5-F), augmented with a 5′ KpnI site (underlined in Table 1) and a common reverse primer (SD-R) containing a XhoI site (underlined in Table 1) were used to obtain the full-length promoter fragment (SD1, 2645 bp) and four truncated fragments (SD2, 1613 bp; SD3, 1136 bp; SD4, 806 bp; SD5, 303 bp). The distance of insert fragments SD1, SD2, SD3, SD4 and SD5 to the putative transcription start site (TSS) +1, assumed to be the first base of the first non-coding exon, was −1856 bp, −824 bp, −347 bp, −17 and +481 bp, respectively (Fig. 2). The promoter fragments were amplified using PrimeSTAR Master Mix (Takara, Japan). PCR products were purified using General DNA Purification Kit (Tiangen, China), digested with KpnI-HF and XhoI, and inserted into pGL4.10 [luc2] (Promega, USA) vector digested with the same restriction enzymes. Recombinant plasmids were isolated using High Pure Plasmid Extraction kit (Roche, Switzerland), and construction were verified by sequencing in Sangon Biotech (Sangon Corporation, China).

**Table 1:** Primers used for creating deletion constructs of *Epinephelus coioides fads*2 promoter.

Primer name	Primer sequence (5′-3′)	Fragment (bp)
SD1-F	GGGGTACCTTTCATCTTGACCGTGTTGG	2646
SD2-F	CCGGTACCGCAGACTGACTTGGCAGAGAT	1614
SD3-F	GGGGTACCAATGGTAATGTGCAGTAACAC	1137
SD4-F	GGGGTACCGTGGGTGAATGAGTCCGTGA	810
SD5-F	GGGGTACCTGCGCCCTGCTTCACCAG	310
SD-R	CCGCTCGAGCCTCACTGCTGCCTCTGG	

### Site-directed mutagenesis of *E. coioides fads*2 promoter

Mutations of *E*. *coioides fads*2 promoter were performed with Muta-direct^TM^ site-directed mutagenesis kit (SBS Genetech, China) according to the manufacturer’s protocol, and were confirmed by sequencing. The construct SD3 including the core promoter region was used as wildtype for mutations experiments. TRANSFAC^®^, MatInspector^®^ and JASPAR^®^ were used to predict potential TF binding sites on *E*. *coioides fads*2 promoter. Ten TF binding sites were found (Fig. 4), and thus site-directed mutations were produced aiming at these TF binding sites, details were showed in Table 2. Similarly, two bases “A” on sequence of *E*. *coioides fads*2 promoter corresponding to Sp1 binding site of *S*. *canaliculatus fads*2 (Δ6/Δ5 *fad*) promoter were mutated into “G”, and thus *E*. *coioides fads*2 promoter (SD1, SD2 and SD3) acquired a Sp1 binding site (−165 bp to TSS). The influence of TF binding site mutations or Sp1 mutation on promoter activity of *E*. *coioides fads*2 were measured by Dual luciferase assay as below.

**Table 2:** Primers used for site-directed mutations of TFs on *Epinephelus coioides fads*2 promoter.

TFs	Primer name	Primer sequence (5′-3′)	Mutated sequence
NF1	NF1-F	TATACTGTACGCCGCCAGAAACCCTCGAATG	ATTGGC → ×
	NF1-R	CATTCGAGGGTTTCTGGCGGCGTACAGTATA	
NFY	NFYA-F	GATATACTGTACGCCGCCCAGAAACCCTCGAAT	ATTGG → ×
	NFYA-R	ATTCGAGGGTTTCTGGGCGGCGTACAGTATATC	
HNF4γ	HNF4G-F	CGAATGATCTGCTCGGAATACTGAATGAGTGGGTG	ATTGG → ×
	HNF4G-R	CACCCACTCATTCAGTATTCCGAGCAGATCATTCG	
RXR	RXR-F	GGAATTTGTACTGAATGAGTCCGTGAACATATTAGAC	ATTGG → ×
	RXR-R	GTCTAATATGTTCACGGACTCATTCAGTACAAATTCC	
YY1	YY1-F1	GTAACATACTGTATATAACTGGATAACATACTG	ATAATGG → ×
	YY1-R1	CAGTATGTTATCCAGTTATATACAGTATGTTAC	
	YY1-F2	GGATAACATACTGTATATAACTGGCTAATATTCATGT	ATAATGG → ×
	YY1-R2	ACATGAATATTAGCCAGTTATATACAGTATGTTATCC	
	YY1-F3	CATACTGTATATAACTGGATGATATTCATGTAACATG	ATAATGG → ×
	YY1-R3	CATGTTACATGAATATCATCCAGTTATATACAGTATG	
TBP	TBP-F1	ATGTAACATACTGCATAATGGTGGATAACATACTG	TATATAA → ×
	TBP-R1	CAGTATGTTATCCACCATTATGCAGTATGTTACAT	
	TBP-F2	GGTGGATAACATACTGCATAATGGTGGCTAAT	TATATAA → ×
	TBP-R2	ATTAGCCACCATTATGCAGTATGTTATCCACC	
	TBP-F3	ATGTAACATACTGCATAATGGTGGATGATATTC	TATATAA → ×
	TBP-R3	GAATATCATCCACCATTATGCAGTATGTTACAT	
SP1	Sp1-F	GGCTGTTTCCGTCTGGGCGCGCTGACTGTGAGTAG	GTGAGCGCAC to CTGGGCGCGC
	Sp1-R	CTACTCACAGTCAGCGCGCCCAGACGGAAACAGC	

Notes: Details of binding sites for TFs are shown in Fig. 3. The bases underlined are chosen for site-directed mutant (bases replacement or deletion), “×” means deletion.

### Cell culture, transfection and dual luciferase assay

Tilapia liver cell line HepaT were cultured in Leibovitz’s L15 Medium (Gibco, USA) with 10% fetal bovine serum (Gibco, USA) at 28 °C in a humidified incubator. For DNA transfection, HepaT cells were seeded in 96-well cell culture plates and grown for 24 h to 90% confluent. Transfection were conducted using Lipofectamine^®^ LTX Reagent with PLUS^TM^ reagent (Invitrogen, USA) according to the manufacturer’s instructions. Briefly, 100 ng of promoter reporter plasmid were co-transfected with 10 ng of pGL4.75 into the cells. Each plasmid complex was transfected in triplicate in three independent experiments. Firefly and Renilla luciferase activities were measured using Dual-Glo Luciferase Assay system E2940 (Promega, USA) according to manufacturer’s instructions. Specifically, 24 h after transfection, 75 μL of Dual-Glo Luciferase Assay Reagent was added into each well, the plate was incubated at room temperature for 10 mins. Then the firefly luminescence was measured on a Tecon microplate reader (Tecon, Switzerland), followed by the addition of Dual-Glo Stop & Glo Reagent into the plate. Finally, after incubation at room temperature for 10 mins, Renilla luminescence were measured and the ratio of firefly/renilla luminescence for each well was calculated. The promoter activity was calculated from the chemical luminescence intensity ratio of firefly: renilla luciferase for each construct, and then compared with the activity of vector pGL4.10 luciferase^17^.

### Statistical analysis

All data were presented as mean ± SEM, n = 6 for tissue-specific distribution of *fads*2 mRNA and n = 3 for evaluating the effects of progressive deletions, TF binding site mutations, Sp1 replacement on promoter activity. Data were analyzed suing One Way analysis of variance (ANOVA) followed by Tukey’s multiple comparison test or Student’s t-test with Origin 7.0. A significance of *P* < 0.05 was applied to all statistical tests performed.

## Electronic supplementary material

Supplementary Fig. S1. Sequence of candidate promoter of fads2 in Epinephelus coioides.

## References

[1] Uauy R (2001). Essential Fatty Acids in Visual and Brain Development. Lipids..

[2] Campoy C (2012). Omega 3 fatty acids on child growth, visual acuity and neurodevelopment. Br J Nutr..

[3] Delgado-Lista J (2012). Long chain omega-3 fatty acids and cardiovascular disease: a systematic review. Br J Nutr..

[4] Awada M (2013). n-3 PUFA added to high-fat diets affect differently adiposity and inflammation when carried by phospholipids or triacylglycerols in mice. Nutr Metab..

[5] Delarue J (2004). N-3 long chain polyunsaturated fatty acids: a nutritional tool to prevent insulin resistance associated to type 2 diabetes and obesity?. Reprod Nutr Dev..

[6] Patterson, E. *et al*. Health implications of high dietary omega-6 polyunsaturated Fatty acids. *J Nutr Metab*. 539426 (2012). 10.1155/2012/539426 PMC3335257 22570770

[7] Naylor RL (2009). Feeding aquaculture in an era of finite resources. Proc Natl Acad Sci USA.

[8] Turchini GM, Torstensen BE, Ng W (2009). Fish oil replacement in finfish nutrition. Rev Aquacult..

[9] Tocher DR (2015). Omega-3 long-chain polyunsaturated fatty acids and aquaculture in perspective. Aquaculture..

[10] Vagner M, Santigosa E (2011). Characterization and modulation of gene expression and enzymatic activity of delta-6 desaturase in teleosts: a review. Aquaculture..

[11] Geay F (2012). Characteristics of*fads*2 gene expression and putative promoter in European sea bass *(Dicentrarchus labra*x): comparison with salmonid species and analysis of CpG methylation. Mar Genom..

[12] Li S (2014). Characterization, mRNA expression and regulation of Δ6 fatty acyl desaturase (FADS2) by dietary n−3 long chain polyunsaturated fatty acid (LC-PUFA) levels in grouper larvae (*Epinephelus coioides*). Aquaculture..

[13] Xie D (2015). Characteristics of LC‐PUFA biosynthesis in marine herbivorous teleost *Siganus canaliculatus* under different ambient salinities. Aquacult Nutr..

[14] Xie D (2014). Cloning, functional characterization and nutritional regulation of Δ6 fatty acyl desaturase in the herbivorous euryhaline teleost *Scatophagus argus*. PLoS one.

[15] Wang S (2014). Investigating long-chain polyunsaturated fatty acid biosynthesis in teleost fish: Functional characterization of fatty acyl desaturase (Fads2) and Elovl5 elongase in the catadromous species, Japanese eel *Anguilla japonica*. Aquaculture..

[16] Huang Y (2017). Cloning, tissue distribution, functional characterization and nutritional regulation of Δ6 fatty acyl desaturase in chu’s croaker *Nibea coibor*. Aquaculture..

[17] Zheng X, Leaver MJ, Tocher DR (2009). Long-chain polyunsaturated fatty acid synthesis in fish: Comparative analysis of Atlantic salmon (*Salmo salar L*.) and Atlantic cod (*Gadus morhua L*.) Δ6 fatty acyl desaturase gene promoters. Comp Biochem Physiol B Biochem Mol Biol..

[18] Li Y (2008). The effects of dietary fatty acids on liver fatty acid composition and Δ6-desaturase expression differ with ambient salinities in *Siganus canaliculatus*. Comp Biochem Physiol B Biochem Mol Biol..

[19] Li Y (2010). Vertebrate fatty acyl desaturase with Δ4 activity. Proc Natl Acad Sci USA.

[20] Monroig Ó (2012). Elongation of long-chain fatty acids in rabbitfish *Siganus canaliculatus*: Cloning, functional characterisation and tissue distribution of Elovl5-and Elovl4-like elongases. Aquaculture..

[21] Chen C (2017). Effects of different dietary ratios of linolenic to linoleic acids or docosahexaenoic to eicosapentaenoic acids on the growth and immune indices in grouper. Epinephelus coioides. Aquaculture..

[22] Monroig O (2010). Multiple genes for functional ∆6 fatty acyl desaturases (Fad) in Atlantic salmon (*Salmo salar L*.): gene and cDNA characterization, functional expression, tissue distribution and nutritional regulation. Biochim Biophys Acta..

[23] Jaya-Ram A (2011). Molecular cloning and ontogenic mRNA expression of fatty acid desaturase in the carnivorous striped snakehead fish (*Channa striata*). Comp Biochem Physiol A Mol Integr Physiol..

[24] Mohd-Yusof NY (2010). Investigation of highly unsaturated fatty acid metabolism in the Asian sea bass, *Lates calcarifer*. Fish Physiol Bioche..

[25] Tocher DR (2006). Highly unsaturated fatty acid synthesis in marine fish: cloning, functional characterization, and nutritional regulation of fatty acyl Δ6 desaturase of Atlantic cod (*Gadus morhua* L.). Lipids..

[26] Zuo R (2016). Molecular cloning, tissue distribution and nutritional regulation of a Δ6-fatty acyl desaturase-like enzyme in large yellow croaker (*Larimichthys crocea*). Aquac Res..

[27] Monroig O (2009). Expression of long-chain polyunsaturated fatty acid (LC-PUFA) biosynthesis genes during zebrafish *Danio rerio* early embryogenesis. Biochim Biophys Acta..

[28] Seiliez I (2001). Cloning, tissue distribution and nutritional regulation of a Δ6-desaturase-like enzyme in rainbow trout. Comp Biochem Physiol B Biochem Mol Biol..

[29] Bell JG (2001). Replacement of fish oil with rapeseed oil in diets of Atlantic salmon (Salmo salar) affects tissue lipid compositions and hepatocyte fatty acid metabolism. J Nutr..

[30] Tocher DR (2010). Fatty acid requirements in ontogeny of marine and freshwater fish. Aquac Res..

[31] O’Malley BW, Towle HC, Schwartz RJ (1977). Regulation of gene expression in eucaryotes. Annu Rev Genet..

[32] Dong Y (2016). Hepatocyte Nuclear Factor 4α (HNF4α) Is a Transcription Factor of Vertebrate Fatty Acyl Desaturase Gene as Identified in Marine Teleost *Siganus canaliculatus*. PLoS one..

[33] Chung HH, Zulkharnain A (2016). Molecular cloning of a functional Fads2 promoter fromZebrafish. J Biochem Microbiol Biotechn..

[34] Tang C (2003). Regulation of human Δ-6 desaturase gene transcription: identification of a functional direct repeat-1 element. J Lipid Res..

[35] Amemiya-Kudo M (2002). Transcriptional activities of nuclear SREBP-1a, -1c, and-2 to different target promoters of lipogenic and cholesterogenic genes. J Lipid Res..

[36] Teran-Garcia M (2007). Polyunsaturated fatty acid suppression of fatty acid synthase (FASN): evidence for dietary modulation of NF-Y binding to the *Fasn* promoter by SREBP-1c. Biochem J..

[37] Nara TY (2002). The E-box like sterol regulatory element mediates the suppression of human Δ-6 desaturase gene by highly unsaturated fatty acids. Biochem Biophys Res Commun..

[38] Dong X (2017). Regulation of FADS2 transcription by SREBP-1 and PPAR-α influences LC-PUFA biosynthesis in fish. Sci Rep-UK..

[39] Daigo K (2011). Proteomic analysis of native hepatocyte nuclear factor-4α (HNF4α) isoforms, phosphorylation status, and interactive cofactors. J Biol Chem..

[40] Pan G (2017). PATZ1 down-regulates FADS1 by binding to rs174557 and is opposed by SP1/SREBP1c. Nucleic Acids Res..

[41] Chen S (2018). Fatty Acid Elongase-7 is Regulated Via SP1 and is involved in lipid accumulation in bovine mammary epithelial cells. J. Cellular Physiol..

[42] Livak KJ, Schmittgen TD (2001). Analysis of relative gene expression data using real-time quantitative PCR and the 2^−ΔΔCT^ method. Methods..

