# Supplementary material for: Characteristics of the fads2 gene promoter in marine teleost Epinephelus coioides and role of Sp1-binding site in determining promoter activity
Source: Sci Rep. 2018 Mar 28;8:5305. doi: 10.1038/s41598-018-23668-w (PMC5871817; doi:10.1038/s41598-018-23668-w)
Supplement: Supplementary file 1 — Supplementary Fig. S1. Sequence of candidate promoter of fads2 in Epinephelus coioides. [file 41598_2018_23668_MOESM1_ESM.docx]

**Title**

Characteristics of the *fads*2 gene promoter in marine teleost *Epinephelus coioides* and role of Sp1-binding site in determining promoter activity

**Authors**

Dizhi Xie^1^**^†^**, Zhixiang Fu^2^**^†^**, Shuqi Wang^2^, Cuihong You^2^, Óscar Monroig^3^, Douglas R. Tocher^3^, Yuanyou Li^1^*

TTTCATCTTGACCGTGTTGGTCAATGTAAAAGGGGGAGAAAAAAGCCACATTAAATCTTC -1797

TCTGCACAAAAAAAGAGTAAATAAAAGTTTCAACTCTACATAGATAGATGGATAGATTTT -1737

CTGTACCAGGCTGTAAACATGTTTGTTTCTGCAGTACAGTTTGGCATTTTAACATAGGGG -1677

TCTATGATGATTGACTCACTTTTGGAGTTAGCGCCAAGTGGCCATTTAAGGAACTGCACC -1617

TTTGTCACGTCCATGTTGACTTCATTTTCAGCCCTAGAGGTTGCTGCTTGGATGTACTAC -1557

AGTGCATGCATTTTGTGTGTCCAAGTGGTGTTGCTTTTACCCCAGCAGAACACAGCCCAC -1497

AGGCTCATATTTAATTAATGTTAATGCTTTTATTACATCATATTTTCCAGATAGCCCACA -1437

ATAAGCTTAAGTAAAGTGTTAATCATTTATGTAATGCTGTATAATTTATATAGAGGGTAG -1377

TGCCTTTCATTTATTGCATATTTGAAAAGTATACTGTATGACTGTACTTGCTTATGATAA -1317

ATTATATGATATAGCAAGGAAAATATAAAAAAAAAGGAACGGTCACAAAGACAGTGAAGG -1257

GGTTTGAGGTGGCAACCTTTGGTGTCAGTTCTACGGGAGTCAGTGGGGCGGGAACTGCAA -1197

ATCTCACTATAACCCATCAAATCTTTGTTATTTCAACTTTGACCGAAGAATCCTTCCACT -1137

AGATTAAAAAACACACATTATGTGATGCTAACAAATTTTTTGTCATTGTCAACGATAATA -1077

AAGTCCCAGTGTCCTCAAACGAGACGACAATAAAGTCCCAATGTCCTCAAACGAGACGAT -1017

AATTAAGTCCCAGTGTCTTAAAACAAGTACTTCCTAACTAACAGTGTCTTAGCCTGTTAG -957

TGTTACTTCTTACTAAAATTGGTGAAATTTGCTGCTAAATCAAACTACAAACATTATTAC -897

TCATAAATAAACAAGTGTCAACCATAAAATGTGATGTTTTGGACCTTGTCCACTTTTGTG -837

TTGGGATTGACTGCAGACTGACTTGGCAGAGATGGCTGCCATCTTGTTTTTACATGGATT -777

AGTAGATATTGTGCTCTTGCTACCACTAGACTGCACGAAAAAGTGTCCACGAATGAGGAC -717

AACAGGTCTGAGTTAAGTACAAAGAAGTATAAGGTCAAGTAAACCCAAATTGTGATGTCC -657

ATATATGAGGACACAGGGTCTCTGGAGGATATATATGCTTACACTGGCCTTAATCAGTGA -597

CAAGATGTGTTGTCCCAATCACCCAATTGGGGTCAGTTTACATAAAAAGGGTATGTCATG -537

TCTTCTTGGAATATGATAGAAAATTATATCCTCCTTAAATGGACAGGTAACATCTTCAGT -477

ATTACACACATGTATCATTTGTTGTTGTTTTTTAAATTTATGGCAAGAAAGCTAACAGAG -417

GGAGGAAATTCACCCTTAATTCACCCTACTAAAATAACCACTATGATCATATTTAATGTT -357

GTAGGCTACAATGGTAATGTGCAGTAACACTGTTATAACATTTATGTAACATACTGTATA -297

TAACATAATGGTGGATAACATACTGTATATAACATAATGGTGGCTAATATTCATGTAACA -237

TACTGTATATAACATAATGGTGGATGATATTCATGTAACATGGTGGCTGGTGTGTGAGCG -177

GCTGTTTCCGTGTGAGCGCACTGACTGTGAGTAGACTGTGTGATATTCAGACGAGCTGCT -117

TGTTGAGCCTATTGCACATCAGCCAGCGGTCCAGGATATACTGTACGCCGATTGGCCCAG -57

AAACCCTCGAATGATCTGCTCGGAATTTGTACTGAATGAGTGGGTGAATGAGTCCGTGAA +4

CATATTAGACAAGACAAGAGGATGAATTTTGGGATGTTTACTATTGGAAATGACTGTGAC +64

CGGAGCGGGAGCAGATAAAATGAGCCGGTGTGTCAGTGTGACTGAAACCAAGGGAGAGTA +124

GCCAAAATCTGGATACTGTAAGTGTGAAATATTTTTCATCTTTCCATGCAGCGGGCCTGC +184

ACAGCCCGCCAGCCGCTCTCTGCGATTACTGTCCAACTGATCAATACGCGGGTGATAAAC +244

GTTAATCGATAGGCAGTTTGTGGTTTCATGGGTCTGATTTGTGTCCAGTTTTTGCGCATC +304

AACTGCTTTTTCGTGTGATGAAATGCGACTTATTGCATAAATGCGCTGCTATGGAAATGA +364

ACAGTCGCACAAAAACGCACCTACAGGCTAAATATAGATTATTTCCAGTGTTTCTATCAT +424

GCGGCATGTGTATCAAAGGTTAATGTTTATACAAACAAGTGATCCATATGTGAAAATGCG +484

CCCTGCTTCACCAGCAGCCTCCTGTTAGTCGGATTTCATCATCAATTATTAGTGGGGATG +544

TTGCTCTGCTCTTTGTCTGACTTCTCTTCTGTCACAATGAGTTGGGTGGTTAAAGCAAAG +604

AGAGCAACAGGTTAAAACACTCTTCCTCTCATTGCTGCTCCCTCTGCACAATCTGTGACT +664

GTACACTGCCAGACAGGGGCGTTAAACACTCCTTCTTCTTTCTGTCTGTAACATCCAGGT +724

GTCAAACTTTGTGGCTGACTGGCCCTTTAGTGTGCAGGTGGACCCAGGCCAGAGGCAGCA +784

GTGAGGATG

**Supplementary Fig. S1. Sequence of candidate promoter of *fads*2 in *Epinephelus coioides.*** Gene sequence is numbered relative to the first base of the transcription start site (TSS), assumed to be the first base of the 5′ non-coding exon. Exon sequences are marked in grey background. ATG start codon was shown in bold
